# Association Between Social Determinants of Health and Readmission Outcomes in Heart Failure: A Retrospective Cohort Study Using Medical Information Mart for Intensive Care IV (MIMIC-IV)

**DOI:** 10.7759/cureus.111435

**Published:** 2026-06-24

**Authors:** Moses C Odoeke, Favour K Mbakwe, Jemie U Nnanna, Vincent U Barrah, Jennifer C Mbonu, Steve Okwu, Anulika Anyata, Obinna Abonyi, Akinyele Oladimeji

**Affiliations:** 1 Internal Medicine, University of Toledo, Toledo, USA; 2 Internal Medicine, Obafemi Awolowo University, Osun, NGA; 3 Internal Medicine, Bingham University College of Health Sciences, Jos, NGA; 4 Hospital Medicine, Mercy Medical Center Cedar Rapids, Cedar Rapids, USA; 5 Otolaryngology-Head and Neck Surgery, University of Illinois Chicago, Chicago, USA; 6 Social Work/Medicine and Surgery, Hennepin Healthcare, Crimean Federal University, Minneapolis, USA; 7 Cardiothoracic Surgery, Beacon Hospital, Dublin, IRL; 8 Community Medicine, Alex Ekwueme Federal University Ndufu-Alike, Abakaliki, NGA; 9 General Practice, University of West Indies, Kingston, JAM; 10 Family Medicine, Alberta Health Services, Edmonton, CAN

**Keywords:** health disparities, heart failure, intensive care, mimic iv, readmission, social determinants of health

## Abstract

Background: Heart failure remains a major contributor to hospital readmissions and healthcare burden in the United States. Social determinants of health may influence post-discharge outcomes, yet their role among critically ill patients is not fully characterized.

Objective: To evaluate the association between selected social determinants of health and 30-day readmission among critically ill patients with heart failure.

Methods: This retrospective cohort study used the Medical Information Mart for Intensive Care IV (MIMIC IV) version 3.1 database. Adult patients with heart failure admitted to the intensive care unit were identified using diagnosis codes. The primary outcome was 30-day hospital readmission. Exposures included insurance type, marital status, and race. Multivariable logistic regression was performed, adjusting for age, sex, Charlson Comorbidity Index, Sequential Organ Failure Assessment score, and length of stay.

Results: A total of 16,324 patients were included. The 30-day readmission rate was 17.6%. Black patients had higher odds of readmission compared to White patients, aOR: 1.37, 95% CI: 1.21 to 1.56, p<0.001, and Hispanic patients also had higher odds, aOR: 1.31, 95% CI: 1.04 to 1.64, p=0.020. Private insurance was associated with lower odds compared to Medicare (aOR: 0.83, 95% CI: 0.73 to 0.95, p=0.008). A higher Charlson Comorbidity Index and longer length of stay were associated with increased odds of readmission, while a higher Sequential Organ Failure Assessment score was associated with lower odds.

Conclusion: This study highlights that social and clinical factors are associated with 30-day readmission in critically ill patients with heart failure. Consideration of social context alongside clinical risk may improve identification of patients at higher risk of rehospitalization.

## Introduction

Heart failure (HF) is a leading cause of morbidity and mortality worldwide and contributes significantly to healthcare expenditure [1]. Hospital readmission following discharge from a hospital presents a continuing problem for healthcare systems because many patients will return to the hospital soon after discharge, post-index admission, despite advancements in therapeutic management [2,3]. The persistence of this pattern indicates that the high rate of readmission cannot be attributed solely to the clinical severity of the patient [4]. The designation of a 30-day readmission rate is now widely utilized as an indicator of the quality of care and transition assistance provided to patients by the healthcare system [5,6].

HF readmissions are influenced by multiple clinical and non-clinical factors. Post-discharge stability influenced by a successful post-discharge period will be affected by their clinical instability, the number of comorbid diseases they have, their discharge plan, access to medications, and provision of follow-up care from outpatient providers [7,8]. Social determinants of health (SDOH) represent economic and social situations that will influence a patient’s ability to prevent illness and a patient’s ability to access healthcare, as well as their ability to manage their chronic illnesses [9,10]. In populations where large datasets are available, the predominant variables considered to reflect a person’s access to healthcare, social support, and structural inequity are insurance type, marital status, and ethnicity [11,12]. These same variables may have a direct impact on a patient’s ability to afford medications, attend follow-up appointments, understand their discharge instructions, and obtain immediate care for exacerbated symptoms [13,14].

Ongoing differences in terms of HF readmissions indicate that patients who experience high levels of social vulnerability may be at increased risk for readmission, despite being treated in the same healthcare organization [15]. Patients receiving public payments for their medical services (Medicaid), those who have low-income levels, and, ultimately, patients with no medical insurance are subject to financial constraints and limited access to transition supports upon being discharged from the hospital [13,14]. Patients who are single or unmarried may lack informal support systems to assist them with adhering to their medication regimens, keeping track of their dietary intake, and identifying symptoms of worsening HF, all of which are essential for successful self-management of HF [15-17]. Ethnic-minority patients may find themselves disadvantaged in terms of accessing outpatient (after-hospital) care and communicating with their care provider, as well as having to deal with systemic inequities in how healthcare is delivered [16,18]. Therefore, these mechanisms indicate that the readmission risk experienced by patients after being discharged from the hospital reflects both their biological vulnerabilities and the inequitable circumstances surrounding their recovery from acute illness after being discharged from a hospital.

While some previous studies have linked the SDOH to readmission among patients with HF, there are still significant gaps in current knowledge concerning this important area of research [9,11]. Most of the studies have relied exclusively on data from single-center cohorts, as well as general hospitalized patients, whereas critically ill HF patients have not been studied very much. As a result, the extent to which SDOH are related to readmission outcomes of these high-risk patients remains unknown [19]. The MIMIC-IV database contains large and completely publicly available datasets that provide comprehensive real-world data from intensive care units (ICUs) and are appropriate for conducting a retrospective cohort analysis of readmission outcomes for patients with HF [19].

Previous studies have demonstrated associations between social determinants of health and readmission among general heart failure populations; however, evidence remains limited among critically ill patients requiring intensive care unit admission. Because insurance status, marital status, and race are commonly used indicators of healthcare access, social support, and structural inequities in large administrative datasets, they were selected as proxy measures of social determinants of health in the present study. The primary objective of this study was to evaluate the association between these social determinants of health and 30-day readmission among critically ill patients with heart failure using MIMIC-IV data.

## Materials and methods

Study design and data source

This study was a retrospective cohort analysis conducted using the Medical Information Mart for Intensive Care IV (MIMIC-IV) version 3.1 database, a large publicly available critical care dataset containing deidentified health-related data from patients admitted to intensive care units at Beth Israel Deaconess Medical Center [20]. Access to the database was obtained after completion of the required certification process, credentialing, and data use agreement through PhysioNet. Therefore, all conditions for data access and use were fulfilled prior to analysis, and no additional license was required. Data were accessed through Google BigQuery. Structured query language was used to extract patient-level data from hospital and intensive care unit tables, including admissions, patients, diagnoses, and derived clinical scores.

Study population

The study population included adult patients aged 18 years or older with a diagnosis of heart failure who were admitted to an intensive care unit. Heart failure was identified using International Classification of Diseases, Ninth (ICD-9) Revision codes beginning with 428 and Tenth (ICD-10) Revision codes beginning with I50, as recorded within the MIMIC IV database [20]. Patients were required to have at least one intensive care unit stay linked to a qualifying hospital admission. To avoid duplication and within-patient correlation arising from multiple admissions, only the first intensive care unit stay per patient was retained using chronological ordering of admission time. This approach has been commonly applied in MIMIC-based cohort studies to ensure independence of observations and simplify the interpretation of patient-level outcomes. However, it may exclude patients with recurrent heart failure admissions who are at particularly high risk of readmission and adverse outcomes. The primary outcome was defined using subsequent hospital admissions within 30 days of discharge. After cohort construction, the dataset included 16,622 unique patients. Following exclusion of patients with missing Sequential Organ Failure Assessment (SOFA) scores, the final analytic sample consisted of 16,324 patients.

Variables and measures

The primary outcome was 30-day hospital readmission, defined as a subsequent hospital admission occurring within 30 days of discharge from the index hospitalization. This was derived by ordering admissions chronologically within each patient and calculating the time interval between discharge and the next admission. The main exposures were proxies of social determinants of health, including insurance type, marital status, and race. Insurance type was extracted from the admissions table and categorized into Medicare, Medicaid, private, and self-pay. Marital status was categorized as married, single, divorced, widowed, and other based on recorded values. Race was standardized using predefined string-matching rules applied to the race variable within the admissions table. Categories were grouped as White, Black, Hispanic, Asian, and Other to improve interpretability and maintain sufficient sample sizes for analysis. Demographic variables included age at admission and sex. Clinical covariates included the Charlson Comorbidity Index, which was obtained from the MIMIC-IV-derived comorbidity tables, which use validated ICD-based algorithms to summarize baseline comorbidity burden [21], and the Sequential Organ Failure Assessment score, measured during the first 24 hours of intensive care unit admission and representing severity of illness [22]. Length of stay was calculated as the difference in days between hospital admission and discharge times. All variables were extracted using structured query language and subsequently processed and recoded in StataCorp. 2025. Stata Statistical Software: Release 18. College Station, TX: StataCorp LLC. [24].

Missing data

Missing data were minimal across variables. There were no missing values for the outcome, age, sex, race category, insurance category, marital status, Charlson Comorbidity Index, or length of stay, each with 0.00% missingness. The SOFA score had 1.79% missing data. Given the low proportion of missingness and the role of SOFA as a key clinical covariate, a complete case approach was applied, and observations with missing SOFA values were excluded from the final analysis.

Statistical analysis

Descriptive statistics were used to summarize baseline characteristics of the study population, stratified by 30-day readmission status. Continuous variables were reported using means and standard deviations, and comparisons between groups were performed using independent sample t-tests. Categorical variables were summarized using frequencies and column percentages, and comparisons were conducted using chi-square tests. Multivariable logistic regression analysis was performed to evaluate the association between social determinants of health and 30-day readmission. The model included age, sex, race category, insurance category, marital status, Charlson Comorbidity Index, SOFA score, and length of stay as covariates. Results were reported as odds ratios with 95% confidence intervals. Multicollinearity among independent variables was assessed using variance inflation factors, and VIF values ranged from 1.02 to 1.91, with a mean VIF of 1.25, indicating no significant multicollinearity [23]. All analyses were conducted using StataCorp. 2025. Stata Statistical Software: Release 18. College Station, TX: StataCorp LLC. [24].

Ethical considerations

The MIMIC IV database contains deidentified patient data and is publicly available to researchers who complete required training and data use agreements. As the dataset does not contain identifiable patient information, this study was exempt from institutional review board approval. All analyses were conducted in accordance with the data use agreement and relevant ethical standards for research using secondary deidentified data.

## Results

Table 1 below presents the baseline characteristics of the study population stratified by 30-day readmission status.

**Table 1:** Baseline Characteristics by 30-Day Readmission Status (n=16324) Values are presented as mean (SD) for continuous variables and n (column percent) for categorical variables. Continuous variables were compared using independent sample t tests, and categorical variables were compared using chi-square tests. SOFA refers to the Sequential Organ Failure Assessment (SOFA) score. The asterisk (*) indicates statistical significance at the 5% level of significance. (–) Intentionally left blank. The table was generated by co-authors using Stata version 18 [24].

Variable	No Readmission (n = 13,444)	Readmission (n = 2,880)	Test Statistic	p-value
Age, years, mean (SD)	71.50 (13.56)	70.34 (13.48)	t = 4.14	<0.001*
Charlson Comorbidity Index, mean (SD)	6.85 (2.62)	7.07 (2.55)	t = -4.04	<0.001*
SOFA score, mean (SD)	5.86 (3.71)	5.42 (3.34)	t = 5.91	<0.001*
Length of stay, days, mean (SD)	11.39 (11.64)	13.31 (13.02)	t = -7.87	<0.001*
Gender, n (%)	–	–	χ² (1)= 2.35	0.125
Male	7,511 (55.87%)	1,564 (54.31%)	–	–
Female	5,933 (44.13%)	1,316 (45.69%)	–	–
Race, n (%)	–	–	χ² (4)= 237.69	<0.001*
White	9,117 (67.81%)	2,048 (71.11%)	–	–
Black	1,243 (9.25%)	420 (14.58%)	–	–
Hispanic	353 (2.63%)	109 (3.78%)	–	–
Asian	299 (2.22%)	78 (2.71%)	–	–
Other	2,432 (18.09%)	225 (7.81%)	–	–
Insurance, n (%)	–	–	χ² (3) = 7.84	0.050
Medicare	9,763 (72.62%)	2,124 (73.75%)	–	–
Medicaid	1,291 (9.60%)	299 (10.38%)	–	–
Private	2,076 (15.44%)	405 (14.06%)	–	–
Self-pay	314 (2.34%)	52 (1.81%)	–	–
Marital status, n (%)	–	–	χ² (4)= 196.60	<0.001*
Married	5,828 (43.35%)	1,271 (44.13%)	–	–
Single	2,788 (20.74%)	732 (25.42%)	–	–
Divorced	947 (7.04%)	233 (8.09%)	–	–
Widowed	2,543 (18.92%)	581 (20.17%)	–	–
Other	1,338 (9.95%)	63 (2.19%)	–	–

The results indicate that patients without readmission were slightly older than those who were readmitted, with mean ages of 71.50 (13.56) years and 70.34 (13.48) years, respectively, and this difference was statistically significant (p<0.001). Patients who were readmitted had a higher Charlson Comorbidity Index compared to those without readmission, 7.07 (2.55) versus 6.85 (2.62), and this difference was statistically significant (p<0.001). The mean SOFA score was lower among readmitted patients at 5.42 (3.34) compared to 5.86 (3.71) in those without readmission, and this difference was statistically significant (p<0.001). Length of stay was longer among readmitted patients, with a mean of 13.31 (13.02) days compared to 11.39 (11.64) days in those without readmission, and this difference was statistically significant. There was no statistically significant difference in gender distribution between groups, with males accounting for 7,511 (55.87%) of patients without readmission and 1,564 (54.31%) of those readmitted, and females accounting for 5,933 (44.13%) and 1,316 (45.69%), respectively. Race distribution differed significantly between groups. Among patients without readmission, White individuals comprised 9,117 (67.81%), while among those readmitted, White individuals comprised 2,048 (71.11%). Black patients accounted for 1,243 (9.25%) in the non-readmission group and 420 (14.58%) in the readmission group. Hispanic patients accounted for 353 (2.63%) and 109 (3.78%), Asian patients, 299 (2.22%) and 78 (2.71%); and other races, 2,432 (18.09%) and 225 (7.81%), respectively. The difference between insurance types was not statistically significant (p=0.05). Medicare was the most common category, with 9,763 (72.62%) among those without readmission and 2,124 (73.75%) among those readmitted. Medicaid accounted for 1,291 (9.60%) and 299 (10.38%); private insurance for 2,076 (15.44%) and 405 (14.06%); and self-pay for 314 (2.34%) and 52 (1.81%), respectively. Marital status differed significantly between groups. Married individuals accounted for 5,828 (43.35%) of those without readmission and 1,271 (44.13%) of those readmitted. Single individuals accounted for 2,788 (20.74%) and 732 (25.42%); divorced for 947 (7.04%) and 233 (8.09%); widowed for 2,543 (18.92%) and 581 (20.17%); and other for 1,338 (9.95%) and 63 (2.19%), respectively.

Table 2 presents the results of the multivariable logistic regression analysis examining factors associated with 30-day readmission.

**Table 2:** Multivariable Logistic Regression for 30-Day Readmission Reference categories were male for gender, white for race, Medicare for insurance, and married for marital status. The asterisk (*) indicates statistical significance at the 5% level of significance. (–) Intentionally left blank. The table was generated by co-authors using Stata version 18 [24].

Variable	Adjusted Odds Ratio (95% CI)	p-value
Age (per year increase)	0.99 (0.99–0.99)	<0.001*
Gender	–	–
Female vs. Male	1.03 (0.94–1.12)	0.570
Race	–	–
Black vs. White	1.37 (1.21–1.56)	<0.001*
Hispanic vs. White	1.31 (1.04–1.64)	0.020*
Asian vs. White	1.18 (0.91–1.53)	0.207
Other vs. White	0.59 (0.51–0.69)	<0.001*
Insurance	–	–
Medicaid vs. Medicare	0.90 (0.77–1.05)	0.163
Private vs. Medicare	0.83 (0.73–0.95)	0.008*
Self-pay vs. Medicare	0.75 (0.55–1.02)	0.063
Marital Status	–	–
Single vs. Married	1.06 (0.95–1.19)	0.260
Divorced vs. Married	1.03 (0.88–1.21)	0.701
Widowed vs. Married	1.04 (0.92–1.17)	0.502
Other vs. Married	0.32 (0.24–0.42)	<0.001*
Charlson Comorbidity Index	1.04 (1.03–1.06)	<0.001*
Sequential Organ Failure Assessment score	0.96 (0.95–0.97)	<0.001*
Length of stay (days)	1.01 (1.01–1.02)	<0.001*

The results indicate that increasing age was associated with lower odds of readmission (aOR=0.99, 95% CI: 0.99-0.99, p<0.001). Female sex was not significantly associated with readmission compared to males (aOR=1.03, 95% CI: 0.94-1.12, p=0.570). Compared to White patients, Black patients had higher odds of readmission (aOR=1.37, 95% CI: 1.21-1.56, p<0.001), and Hispanic patients also had higher odds (aOR=1.31, 95% CI: 1.04-1.64, p=0.020). Asian patients did not show a statistically significant difference (aOR=1.18, 95% CI: 0.91-1.53, p=0.207), while patients categorized as other races had lower odds of readmission (aOR=0.59, 95% CI: 0.51-0.69, p<0.001). For insurance type, Medicaid was not significantly associated with readmission compared to Medicare (aOR=0.90, 95% CI: 0.77-1.05, p=0.163), while private insurance was associated with lower odds of readmission (aOR=0.83, 95% CI: 0.73-0.95, p=0.008). Self-pay was not statistically significant (aOR=0.75, 95% CI: 0.55-1.02, p=0.063).

For marital status, the single, divorced, and widowed categories were not significantly associated with readmission compared to married individuals, with (aOR=1.06, 95% CI: 0.95-1.19, p=0.260), (aOR=1.03, 95% CI: 0.88-1.21, p=0.701), and (aOR=1.04, 95% CI: 0.92-1.17, p=0.502), respectively. The other category was associated with lower odds of readmission (aOR=0.32, 95% CI: 0.24-0.42, p<0.001).

A higher Charlson Comorbidity Index was associated with increased odds of readmission (aOR=1.04, 95% CI: 1.03-1.06, p<0.001). A higher SOFA score was associated with lower odds of readmission (aOR=0.96, 95% CI: 0.95-0.97, p<0.001). A longer length of stay was associated with higher odds of readmission (aOR=1.01, 95% CI: 1.01-1.02, p<0.001).

**Figure 1:**
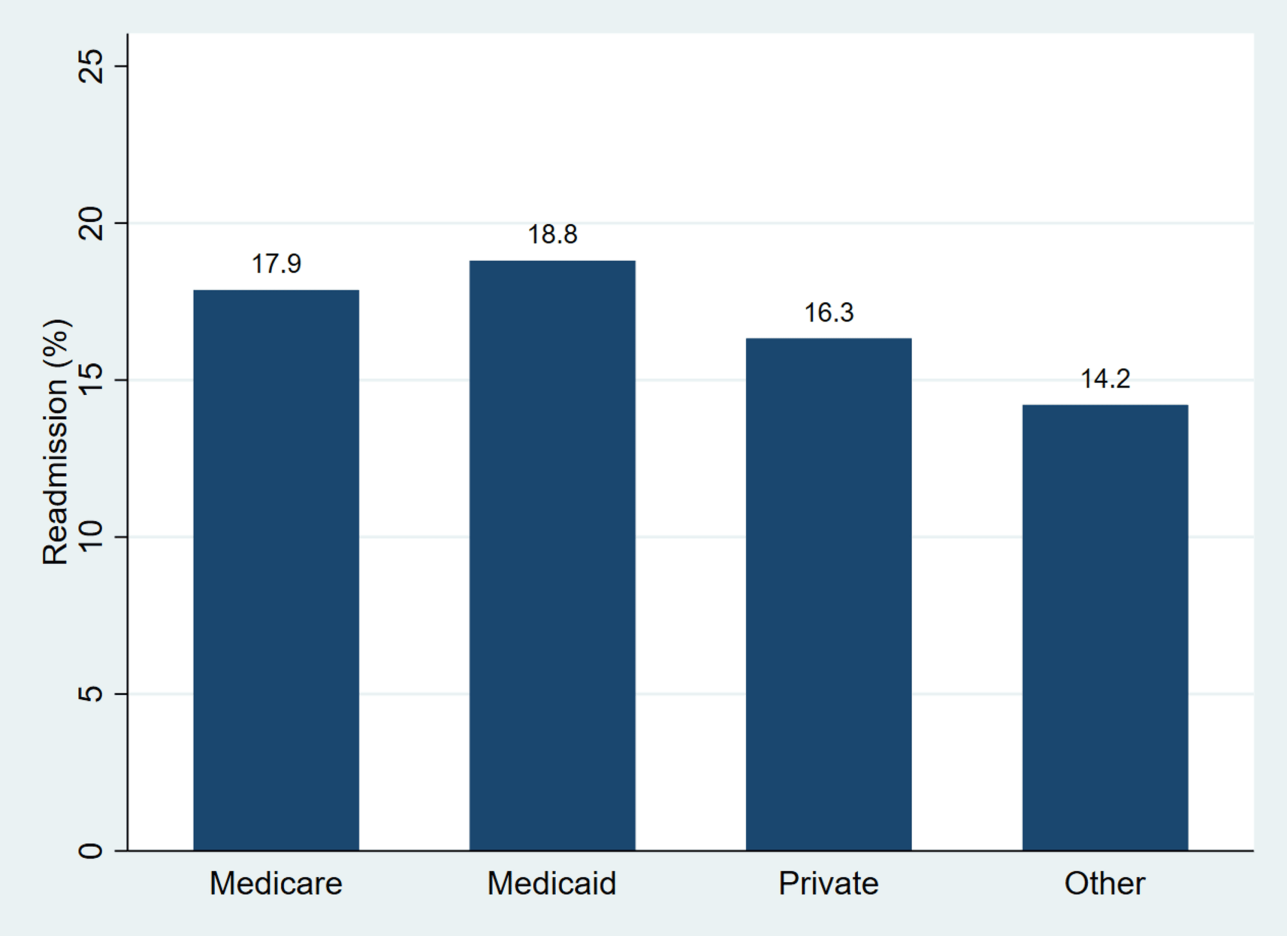
30-Day Readmission by Insurance Type Values represent the proportion of patients readmitted within 30 days expressed as percentages.

Figure 1 illustrates the proportion of patients readmitted within 30 days across different insurance categories.

The results indicate that Medicaid patients had the highest proportion of readmissions, with 299 (18.8%), followed by Medicare with 2,124 (17.9%). Patients with private insurance had a lower proportion at 405 (16.3%), while those categorized as Other had the lowest proportion at 52 (14.2%). Differences between categories are visible, although the magnitude of variation across groups is modest.

## Discussion

The findings of this study address the stated objective by examining the association between selected social determinants of health and 30-day readmission among critically ill patients with heart failure. In this study, the readmission rate was consistent with previously reported patterns in heart failure populations, where early rehospitalization remains frequent and clinically important [3,6]. Differences were observed across social and clinical variables. Higher comorbidity burden and longer hospital stay were associated with increased odds of readmission, which aligns with prior work indicating that patients with greater clinical complexity are more likely to experience recurrent hospitalization [6]. In contrast, higher SOFA scores were associated with lower odds of readmission, a pattern that may reflect differences in survival or discharge pathways rather than a direct protective effect. Social determinants showed measurable variation. Black and Hispanic patients had higher odds of readmission compared to White patients, while patients classified in the Other race category had lower odds. These patterns are consistent with evidence that social and structural factors influence health outcomes beyond clinical characteristics [7,10]. Insurance type also demonstrated variation, with private insurance associated with lower odds of readmission compared to Medicare, while Medicaid and self-pay were not statistically significant. Prior studies have shown that payment source and socioeconomic status are linked to differences in access, continuity of care, and outcomes within the same healthcare system [12-14]. Marital status did not show strong associations except for the Other category, which had lower odds of readmission. This may relate to differences in social support, which has been linked to outcomes in heart failure populations [15]. These findings indicate that both clinical and social factors are associated with readmission risk in this population.

Current United States guidelines for heart failure management emphasize the importance of reducing rehospitalization through coordinated care, early follow-up, and attention to non-clinical factors that influence patient outcomes. The 2022 American Heart Association, American College of Cardiology, and Heart Failure Society of America guideline recommends comprehensive discharge planning, medication optimization, and timely outpatient follow-up as key strategies to reduce readmission [8]. These recommendations are consistent with the patterns observed in this study, where differences in readmission were seen across insurance groups and social categories. The guideline also highlights the role of social determinants, including access to care and social support, in influencing adherence and follow-up, which may contribute to the observed disparities in readmission rates. The present findings support the relevance of these recommendations in critically ill heart failure patients.

Several clinical and contextual mechanisms may explain the observed associations. Patients with higher comorbidity burden may require more complex care and have a higher likelihood of clinical instability after discharge, which increases the chance of readmission [6]. Longer hospital stays may reflect greater illness severity or complications during admission, which can also contribute to subsequent healthcare utilization. The association between lower SOFA scores and higher readmission may reflect that patients with less severe acute illness survive to discharge but remain vulnerable to decompensation, while patients with higher severity may have different clinical trajectories. Social determinants may influence readmission through multiple pathways, including differences in access to outpatient care, medication adherence, and availability of social support [7,10]. Racial and insurance-related differences may also reflect structural inequities in healthcare delivery and resource allocation, which have been documented in prior studies [12,14]. These mechanisms do not imply causation but provide context for the observed associations.

It has been noted that hospital readmissions post-inpatient heart failure treatment are common and can be a marker of poor healthcare quality and efficiency [3]. Clinical decisions and in-hospital treatment, however, are not the only factors influencing readmission, as studies have shown that certain social factors also contribute, including but not limited to level of education, income, insurance type, living situation, race, and social support [7,8,10,12]. The findings from this study go further to provide insight into how both clinical factors and social determinants of health affect readmission. This points to the need for a more comprehensive, patient-centered approach and can, thus, serve as a tool to guide transitional care and discharge planning, helping reduce readmission risks. 

Medication reconciliation is an essential component of discharge planning and safe transitional care [8,24]. Patients with heart failure, especially those who have ended up in the ICU (as our sample group), often have complex drug regimens. Adding comorbidities, which are common in patients with heart failure, increases the likelihood of polypharmacy [8]. This significantly increases the risk of discrepancies, non-adherence, and adverse drug effects. Ensuring the accuracy of discharge medications, addressing affordability, reducing pill burden, and providing clear instructions can reduce these risks, improve continuity of care, and lower readmission rates [25].

Early post-discharge and multidisciplinary follow-up are critical interventions in reducing readmission risk, especially for patients with higher comorbidities or prolonged hospitalization [8]. Early outpatient evaluation allows for reassessment and optimization of medical therapy, provides an opportunity for further education and clarification of new questions or sources of confusion, and enables early identification of clinical deterioration. It is important for providers to schedule outpatient follow-up visits during the discharge process, still taking into consideration the patient’s socioeconomic status (including insurance), comorbidities, and support systems [11,12]. It is also very important to involve the patient in planning this to ensure adherence. Patients with higher comorbidity burden or prolonged hospitalization may benefit from more structured follow-up, including early visits or telehealth monitoring in the first week after discharge.

Strengths and limitations of the study

This study has several strengths and limitations. The use of a large, well-characterized critical care dataset allowed detailed assessment of both clinical and social variables among critically ill patients with heart failure. The analysis included standardized definitions, reproducible data extraction methods, and adjustment for important demographic and clinical covariates.

However, several limitations should be considered. The study relied on routinely collected electronic health record data, which may be subject to misclassification, coding inaccuracies, or incomplete capture of certain variables. Social determinants of health were represented using proxy variables such as insurance type, marital status, and race, which may not fully capture socioeconomic conditions, social support, or broader structural determinants of health. Some variables, including SOFA score, had a small proportion of missing data, although complete case analysis was applied, given the low level of missingness.

To avoid duplication and within-patient correlation, only the first ICU admission per patient was included in the analysis. Consequently, recurrent admissions among the same patient were not evaluated, which may underestimate the burden of readmission among patients with frequently recurring heart failure hospitalizations. Furthermore, because the cohort was restricted to patients admitted to the intensive care unit, the findings may not be generalized to the broader population of hospitalized patients with heart failure, who may differ in disease severity, clinical characteristics, and post-discharge trajectories. The dataset also lacked detailed information on heart failure subtypes, such as heart failure with reduced ejection fraction, mildly reduced ejection fraction, or preserved ejection fraction, which may have different readmission risks and clinical courses.

The observational design limits the ability to infer causal relationships, and residual confounding from unmeasured factors may remain. Variables such as education, income, housing stability, outpatient follow-up, medication adherence, caregiver support, and community-level social determinants were not available in the dataset and may influence readmission risk. In addition, MIMIC-IV is derived from a single tertiary academic medical center, which may limit external generalizability to other healthcare settings and patient populations. Future research should incorporate more comprehensive measures of social context and employ longitudinal approaches that include recurrent admissions. Studies using generalized estimating equations or mixed-effects models may further improve understanding of readmission patterns by accounting for within-patient correlation across multiple hospitalizations.

## Conclusions

This study found that both clinical factors and selected social determinants of health were associated with 30-day readmission among critically ill patients with heart failure. Higher odds of readmission were observed among Black and Hispanic patients, while private insurance was associated with lower odds of readmission compared with Medicare. Greater comorbidity burden and longer hospital stay were also associated with increased readmission risk. These findings suggest that social and clinical characteristics may contribute to variation in readmission outcomes among ICU patients with heart failure. However, given the observational study design and the use of proxy measures for social determinants of health, the results should be interpreted as associations rather than causal relationships. Future research should incorporate more comprehensive measures of social context, evaluate recurrent hospitalizations, and examine these associations in broader heart failure populations to better understand factors influencing readmission risk.

## References

[1] Savarese G, Lund LH (2017). Global public health burden of heart failure. Card Fail Rev.

[2] Virani SS, Alonso A, Aparicio HJ (2021). Heart disease and stroke statistics-2021 update: a report from the American Heart Association. Circulation.

[3] Dharmarajan K, Hsieh AF, Lin Z (2013). Diagnoses and timing of 30-day readmissions after hospitalization for heart failure, acute myocardial infarction, or pneumonia. JAMA.

[4] Ziaeian B, Fonarow GC (2016). Epidemiology and aetiology of heart failure. Nat Rev Cardiol.

[5] Krumholz HM (2013). Post-hospital syndrome--an acquired, transient condition of generalized risk. N Engl J Med.

[6] Desai AS, Stevenson LW (2012). Rehospitalization for heart failure: predict or prevent?. Circulation.

[7] Magnan S (2021). Social determinants of health 201 for health care: plan, do, study, act. NAM Perspect.

[8] Heidenreich PA, Bozkurt B, Aguilar D (2022). 2022 AHA/ACC/HFSA guideline for the management of heart failure: a report of the American College of Cardiology/American Heart Association Joint Committee on Clinical Practice Guidelines. Circulation.

[9] Sterling MR, Ringel JB, Pinheiro LC (2022). Social determinants of health and 30-day readmissions among adults hospitalized for heart failure in the REGARDS study. Circ Heart Fail.

[10] Braveman P, Gottlieb L (2014). The social determinants of health: it's time to consider the causes of the causes. Public Health Rep.

[11] Calvillo-King L, Arnold D, Eubank KJ (2013). Impact of social factors on risk of readmission or mortality in pneumonia and heart failure: systematic review. J Gen Intern Med.

[12] Hu J, Gonsahn MD, Nerenz DR (2014). Socioeconomic status and readmissions: evidence from an urban teaching hospital. Health Aff (Millwood).

[13] Kapoor JR, Kapoor R, Hellkamp AS (2011). Payment source, quality of care, and outcomes in patients hospitalized with heart failure. J Am Coll Cardiol.

[14] Spencer CS, Gaskin DJ, Roberts ET (2013). The quality of care delivered to patients within the same hospital varies by insurance type. Health Aff (Millwood).

[15] Murberg TA, Bru E (2001). Social relationships and mortality in patients with congestive heart failure. J Psychosom Res.

[16] Benjamin EJ, Muntner P, Alonso A (2019). Heart disease and stroke statistics-2019 update: a report from the American Heart Association. Circulation.

[17] Joynt KE, Jha AK (2012). Thirty-day readmissions--truth and consequences. N Engl J Med.

[18] Dharmarajan K, Wang Y, Lin Z (2017). Association of changing hospital readmission rates with mortality rates after hospital discharge. JAMA.

[19] Johnson AE, Bulgarelli L, Shen L (2023). MIMIC-IV, a freely accessible electronic health record dataset. Sci Data.

[20] Johnson A, Bulgarelli L, Pollard T (2024). MIMIC-IV (version 3.1). PhysioNet. RRID:SCR_007345.

[21] Charlson ME, Pompei P, Ales KL, MacKenzie CR (1987). A new method of classifying prognostic comorbidity in longitudinal studies: development and validation. J Chronic Dis.

[22] Vincent JL, Moreno R, Takala J (1996). The SOFA (sepsis-related organ failure assessment) score to describe organ dysfunction/failure. Intensive Care Med.

[23] O’Brien RM (2026). A caution regarding rules of thumb for variance inflation factors. Qua Quan.

[24] (2026). Stata: Homepage. https://www.stata.com/.

[25] Alghamdi DS, Alhrasen M, Kassem A (2023). Implementation of medication reconciliation at admission and discharge in Ministry of Defense Health Services hospitals: a multicentre study. BMJ Open Qual.

